# Retrospective analysis of predictive factors for lymph node metastasis in superficial esophageal squamous cell carcinoma

**DOI:** 10.1038/s41598-021-96088-y

**Published:** 2021-08-16

**Authors:** Rongwei Ruan, Shengsen Chen, Yali Tao, Jiangping Yu, Danping Zhou, Zhao Cui, Qiwen Shen, Shi Wang

**Affiliations:** Department of Endoscopy, Cancer Hospital of the University of Chinese Academy of Sciences (Zhejiang Cancer Hospital), Institute of Cancer and Basic Medicine(IBMC), Chinese Academy of Sciences, Hangzhou, 310022 Zhejiang China

**Keywords:** Gastrointestinal cancer, Oesophagogastroscopy, Risk factors, Oesophageal cancer, Surgical oncology

## Abstract

This study aimed to identify the risk factors of lymph node metastasis (LNM) in superficial esophageal squamous cell carcinoma and use these factors to establish a prediction model. We retrospectively analyzed the data from training set (n = 280) and validation set (n = 240) underwent radical esophagectomy between March 2005 and April 2018. Our results of univariate and multivariate analyses showed that tumor size, tumor invasion depth, tumor differentiation and lymphovascular invasion were significantly correlated with LNM. Incorporating these 4 variables above, model A achieved AUC of 0.765 and 0.770 in predicting LNM in the training and validation sets, respectively. Adding macroscopic type to the model A did not appreciably change the AUC but led to statistically significant improvements in both the integrated discrimination improvement and net reclassification improvement. Finally, a nomogram was constructed by using these five variables and showed good concordance indexes of 0.765 and 0.770 in the training and validation sets, and the calibration curves had good fitting degree. Decision curve analysis demonstrated that the nomogram was clinically useful in both sets. It is possible to predict the status of LNM using this nomogram score system, which can aid the selection of an appropriate treatment plan.

## Introduction

Esophageal cancer is the seventh most common malignancy in the world^1^. Esophageal squamous cell carcinoma confined to intraepithelial (Tis), mucosa (T1a) and submucosal (T1b), irrespective of lymph node metastasis (LNM), is considered to be superficial esophageal squamous cell carcinoma (SESCC) and have a good outcome^2^. Conventional view holds that the standard treatment for most esophageal cancers is radical esophagectomy, even for cancers confined to the mucosa^3^. However, this procedure has high rates of morbidity and mortality^4,5^. Furthermore, surgery may not be carried out in patients who are older or have multiple co-morbidities^6^. In order to reduce surgery-related complications and obtain a higher quality of post-operative life, currently a less invasive treatment method (such as endoscopic resection) has been considered as an alternative to esophageal surgery^7,8^.

Nevertheless, SESCC has LNM potential because the lymphatic-capillary plexus in the mucosa of lamina propria and the submucosa of esophagus are plentiful. As a minimally invasive treatment and do not dissect lymph node, endoscopic resection (ER) is applied to SESCC without LNM. For SESCC with LNM, the proper support is the radical esosophagectomy to remove all potentially involved nodes. Consequently, it is critical to explore the predictive factors of LNM in SESCC patients before ER. In several studies, some imaging methods (EUS, CT or PET) can detect LNM of SESCC, but these methods are not precise enough to completely rule out the presence of the LNM^9–11^. Additionally, the clinicopathological risk factors associated with LNM in SESCC are still understood incompletely^2^.

The purpose of this study was to determine the risk factors of LNM in SESCC patients. Then a nomogram was established using these risk factors, which can help predict LNM and determine whether or not a supplementary esophagus resection is necessary after ER.

## Methods

### Patients selection and data collection

**Figure 1:**
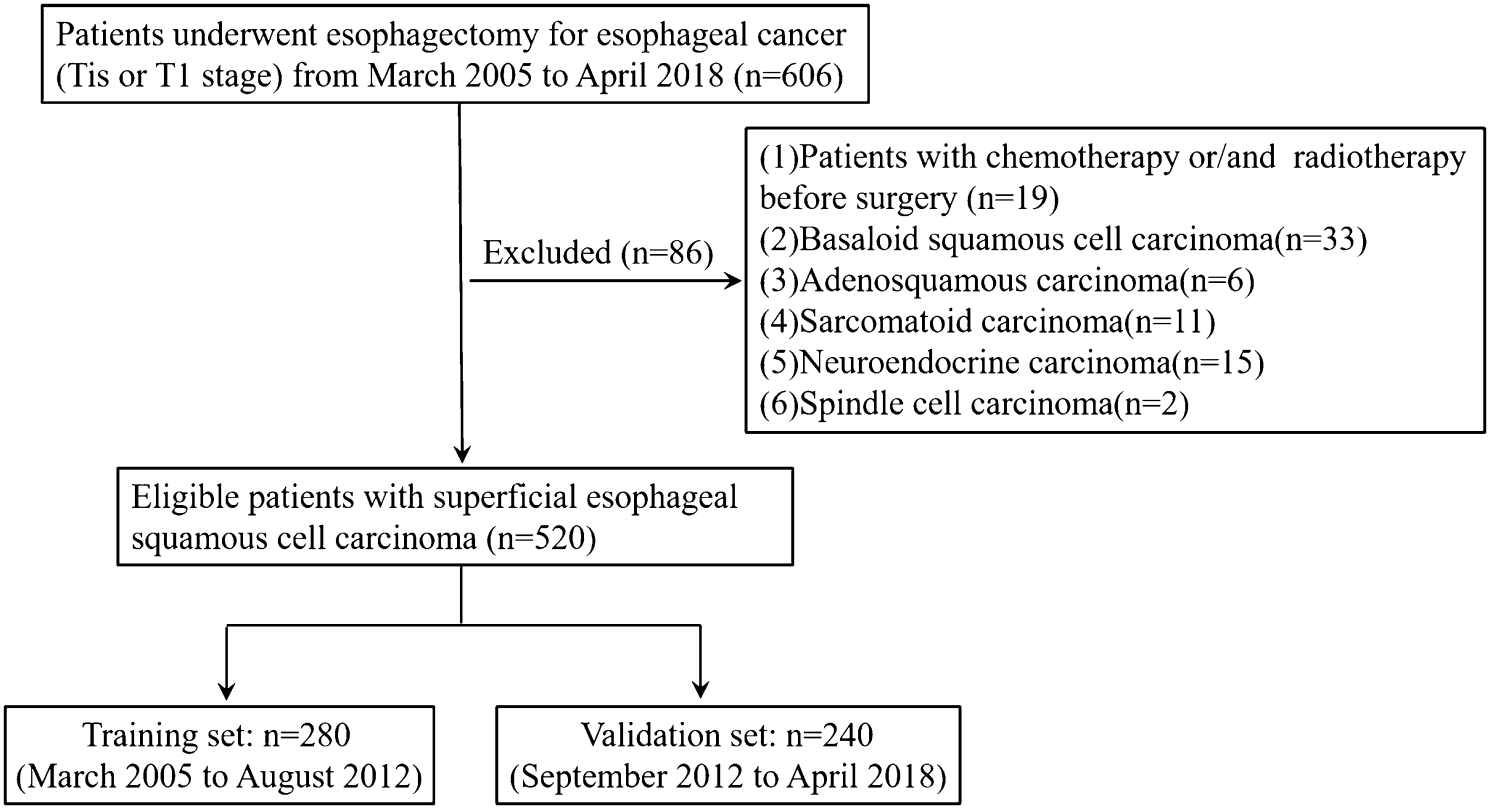
Flowchart of patients included in the analysis.

Between March 2005 and April 2018, the data of patients with histopathologically-confirmed esophageal cancer (Tis or T1 stage) who underwent esophagus resection at Zhejiang Cancer Hospital were retrospectively analyzed. The exclusion criteria were: (1) patients who received chemotherapy or radiotherapy before surgery; (2) patients with basaloid squamous cell carcinoma, adenosquamous carcinoma, sarcomatoid carcinoma, neuroendocrine carcinoma, or spindle cell carcinoma. The final eligible patients with SESCC who were admitted between March 2005 and August 2012 were assigned to the training set and those admitted between September 2012 and February 2018 were assigned to the validation set. Endoscopic findings of the tumors macroscopic type were classified according to the Paris classification^12^. Nonprotruding or nonexcavated superficial tumors were classified as type II (flat type), protruding and excavated superficial tumors were classified as type I and type III (type I and III were considered as nonflat type). The flowchart of patient selection is summarized in Fig. 1.

### Lymph node dissection

In this study, lymph node dissections were performed according to esophageal cancer location^13^. For upper thoracic esophageal cancer, the rate of cervical and upper mediastinal lymph node metastasis is high. Thus, lymph node dissection included the neck (two-field lymph node dissections). For middle thoracic esophageal cancer, lymph node metastasis mainly occurs in the neck and the upper, middle, and lower mediastinum as well as the abdominal cavity. The extent of lymph node dissection included the neck and supraclavicular area (three-field lymph node dissections). For the lower thoracic esophageal cancer, lymph node metastasis mainly occurs in the mediastinum and abdomen, and cervical metastasis is relatively low. So the two-field lymph node dissections were performed for these patients.

### Histopathologic evaluation

Surgical specimens were fixed with formaldehyde and were then cut serially to make slices. The intervals between the tumor tissue and adjacent normal tissues in the slices were 2–5 mm. Tumors that exceed the muscularis mucosa were considered as submucosal invasion^14^. We then classified the location of esophageal cancer according to the guidelines of the American Joint Committee on Cancer^15^. The portion of the esophagus extending from the entrance of the thoracic cavity to the bifurcation of the trachea is considered the upper esophagus, the section from the trachea bifurcation to the distal esophagus (above the esophagogastric junction) is regarded as the middle esophagus, and the intra-abdominal portion of the esophagus and the junction of the esophagus and stomach constituted the lower esophagus.

### Ethics statement

This retrospective study was performed in accordance with the Helsinki Declaration of 1975 and approved by the Ethics Committee of the Zhejiang Cancer Hospital, Hangzhou, China. Written informed consent was obtained from all patients before surgery.

### Statistical analysis

Continuous variables are expressed as median (range) and compared using Mann–Whitney test. Categorical variables were compared using the χ^2^ test or Fisher exact test. All variables associated with LNM at a significant level were candidates for stepwise multivariate logistic analysis. The integrated discrimination improvement (IDI) is the difference in the discrimination slopes for a prediction model with and without one variable, which indicates whether the discrimination slope of a model will improve if one important parameter is added. The net reclassification improvement (NRI) is an index that attempts to quantify how well a new model correctly reclassifies subjects. Typically, this comparison is between an original model and a new model (the original model plus one additional component)^16,17^. The IDI and NRI were calculated using R, version 4.0.3 with the *PredictABEL* package.

According to the results of multivariate logistic regression analysis, we used R software (version 4.0.3) with the *rms* package to formulate a nomogram. The nomogram can proportionally convert each regression coefficient in the logistic regression to a scale of 0–100 points^18^. The points of each independent variable were summed and the predicted probabilities were derived from the total points. The area under the curve (AUC) and calibration curve were used to assess the predictive performance of this nomogram. The most important and final line of evidence for the use of this nomogram is based on the need to interpret individual requirements with regard to additional treatment or care. Decision curve analysis (DCA) offers insight into clinical outcomes on the basis of threshold probability, from which the net benefit could be derived. Net benefit is defined as the proportion of true positives minus the proportion of false positives, weighted by the relative harm of false-positive and false-negative results^19^. In order to evaluate the clinical utility of the nomogram, DCA was performed using R with the *rmda* package. In all analyses, *P* < 0.05 was considered to indicate statistical significance. All analyses were performed using SPSS version 22.0 (SPSS Inc, Chicago, Ill) and R, version 4.0.3.

## Results

### Clinicopathologic characteristics

The clinicopathologic characteristics of the 520 patients are listed in Table 1, and no significant difference was found between training (n = 280) and validation (n = 240) sets. Histopathologically-confirmed LNM was found in 69 (24.6%) and 59 (24.6%) patients in the two sets, respectively. The mean tumor size was 2.87 ± 1.26 cm in training set and 2.93 ± 1.48 cm in validation set. According to the depth of tumor invasion, 62 patients (22.1%) had mucosal cancer and 218 (77.9%) had submucosal cancer in training set. In validation set, 53 patients (22.1%) had mucosal cancer and 187 (77.9%) had submucosal cancer. Lymphovascular invasion (LVI) was found in 29 patients (10.4%) in training set and found in 26 patients (10.8%) in validation set. 33(11.8%) patients in training set underwent 3-field lymph node dissection, and 247(88.2%) underwent 2-field lymph node dissection. In validation set, 21(8.7%) patients underwent 3-field lymph node dissection, and 219 (91.3%) underwent 2-field lymph node dissection. For whole patients (n = 520), paratracheal lymph nodes were the most frequently involved (6.92%), followed by the lesser curvature (6.35%), paracardial nodes (3.27%) and middle paraesophageal (3.27%) (Fig. S1).

**Table 1:** Participant characteristics.

Variables	Training set (n = 280)	Validation set (n = 240)	*P*
**Gender, n (%)**			0.322
Male	235(83.9)	209(87.1)	
Female	45(16.1)	31(12.9)	
Age (years), median (range)	63(25–82)	63(44–79)	0.442
Tumor size(cm), mean ± SD	2.87 ± 1.26	2.93 ± 1.48	0.910
**Circumferential extension, n (%)**			0.146
< 1/4	95(33.9)	81(33.8)	
1/4–2/4	132(47.1)	106(44.2)	
2/4–3/4	42(15.0)	32(13.3)	
> 3/4	11(3.9)	21(8.8)	
**Location within esophagus, n (%)**			0.869
Upper	9(3.2)	9(3.8)	
Middle	189(67.5)	165(68.8)	
Lower	82(29.3)	66(27.5)	
**Depth of invasion, n (%)**			0.987
Mucosa	62(22.1)	53(22.1)	
Submucosa	218(77.9)	187(77.9)	
**Tumor differentiation, n (%)**			0.136
Carcinoma in situ	4(1.4)	12(5.0)	
Well	51(18.2)	41(17.1)	
Moderate	145(51.8)	121(50.4)	
Poor	80(28.6)	66(27.5)	
**LVI, n (%)**			0.887
No	251(89.6)	214(89.2)	
Yes	29(10.4)	26(10.8)	
**Macroscopic type, n (%)**			0.344
I	120(42.9)	102(42.5)	
II	151(53.9)	124(51.7)	
III	9(3.2)	14(5.8)	
**Multiple lesions, n (%)**			0.195
No	262(93.6)	217(90.4)	
Yes	18(6.4)	23(9.6)	
**LNM**			0.987
No	211(75.4)	181(75.4)	
Yes	69(24.6)	59(24.6)	
**Type of lymph node dissection, n (%)**			0.313
3-field	33(11.8)	21(8.7)	
2-field	247(88.2)	219(91.3)	

LVI, lymphovascular invasion; LNM, Lymph node metastasis; I = superficial and protruding type; II = flat type; III = superficial and excavated type; P: Categorical variables—χ^2^ test or Fisher’s exact test; Continuous variables—Mann–Whitney test.

### Independent risk factors of LNM

Comparisons of clinicopathological characteristics between the LNM-positive and-negative groups are summarized in Table 2. In training and validation sets, variables such as tumor size, tumor invasion depth, tumor differentiation, LVI and macroscopic type, were significantly associated with the LNM according to the univariate analysis (Table 2). However, age, gender, circumferential extension, tumor location and the presence of multiple lesions did not show any statistical correlation with LNM. Furthermore, tumor size, tumor invasion depth, tumor differentiation and LVI were identified as independent risk factors of LNM in training and validation sets by multivariate analysis. Interestingly, in training set macroscopic type was not correlated with LNM (*P* = 0.064), while it was considered as a risk factor in validation set (Table 3).

**Table 2:** Clinicopathologic findings according to lymph node metastasis in training and validation sets.

Variables	Training set (n = 280)			Validation set (n = 240)		
	LNM( −) (n = 211)	LNM( +) (n = 69)	*P*	LNM( −) (n = 181)	LNM( +) (n = 59)	*P*
**Gender, n (%)**			0.345			0.274
Male	174(82.5)	61(88.4)		155(85.6)	54(91.5)	
Female	37(17.5)	8(11.6)		26(14.4)	5(8.5)	
Age (years), median (range)	64(25–82)	61(47–81)	0.149	63(44–79)	60(46–76)	0.301
Tumor size (cm), mean ± SD	2.72 ± 1.21	3.33 ± 1.32	** < 0.001**	2.84 ± 1.45	3.22 ± 1.57	**0.046**
**Circumferential extension, n (%)**			0.165			0.180
< 1/4	75(35.5)	20(29.0)		67(37.0)	14(23.7)	
1/4–2/4	101(47.9)	31(44.9)		79(43.6)	27(45.8)	
2/4–3/4	26(12.3)	16(23.2)		21(11.6)	11(18.6)	
> 3/4	9(4.3)	2(2.9)		14(7.7)	7(11.9)	
**Location within esophagus, n (%)**			0.512			0.277
Upper	7(3.3)	2(2.9)		7(3.9)	2(3.4)	
Middle	146(69.2)	43(62.3)		129(71.3)	36(61.0)	
Lower	58(27.5)	24(34.8)		45(24.9)	21(35.6)	
**Depth of invasion, n (%)**			** < 0.001**			**0.001**
Mucosa	57(27.0)	5(7.2)		49(27.1)	4(6.8)	
Submucosa	154(73.0)	64(92.8)		132(72.9)	55(93.2)	
**Tumor differentiation, n (%)**			**0.006**			**0.002**
Carcinoma in situ	3(1.4)	1(1.4)		12(6.6)	0(0)	
Well	43(20.4)	8(11.6)		36(19.9)	5(8.5)	
Moderate	116(55.0)	29(42.0)		93(51.4)	28(47.5)	
Poor	49(23.2)	31(44.9)		40(22.1)	26(44.1)	
**LVI, n (%)**			** < 0.001**			**0.003**
No	201(95.3)	50(72.5)		168(92.8)	46(78.0)	
Yes	10(4.7)	19(27.5)		13(7.2)	13(22.0)	
**Macroscopic type, n (%)**			** < 0.001**			** < 0.001**
I	79(37.4)	41(59.4)		64(35.4)	38(64.4)	
II	128(60.7)	23(33.3)		106(58.6)	18(30.5)	
III	4(1.9)	5(7.2)		11(6.1)	3(5.1)	
**Multiple lesions, n (%)**			0.779			0.804
No	198(93.8)	64(92.8)		164(90.6)	53(89.8)	
Yes	13(6.2)	5(7.2)		17(9.4)	6(10.2)	

The bold values mean statistical significance.

LVI, lymphovascular invasion; LNM, Lymph node metastasis; I = superficial and protruding type; II = flat type; III = superficial and excavated type; P: Categorical variables—χ^2^ test or Fisher’s exact test; Continuous variables—Mann–Whitney test.

**Table 3:** Multivariate logistic analysis of risk factors for lymph node metastasis in training and validation sets.

Factors	Training set			Validation set		
	OR	95% CI	*P*	OR	95% CI	*P*
Tumor size(cm)(continuous)	1.396	1.096–1.778	**0.007**	1.431	1.133–1.806	**0.003**
**Depth of invasion**						
Mucosa	Reference			Reference		
Submucosa	3.112	1.025–9.436	**0.045**	4.384	1.171–16.410	**0.028**
**Tumor differentiation**						
Well or Carcinoma in situ	0.336	0.135–0.839	**0.020**	0.116	0.037–0.367	** < 0.001**
Moderate	0.389	0.196–0.772	**0.007**	0.361	0.175–0.743	**0.006**
Poor	Reference			Reference		
**LVI**						
No	Reference			Reference		
Yes	6.337	2.565–15.659	** < 0.001**	2.905	1.157–7.293	**0.023**
**Macroscopic type**						
Flat (II)	Reference			Reference		
Nonflat (I or III)	1.940	0.962–3.915	0.064	2.346	1.118–4.924	**0.024**

The bold values mean statistical significance.

LVI, lymphovascular invasion; I = superficial and protruding type; II = flat type; III = superficial and excavated type.

### Predictive utility of macroscopic type for LNM prediction

Then model A (including tumor size, depth of tumor invasion depth, tumor differentiation and LVI) was built according to the multivariate logistic analysis results. By adding macroscopic type to the model A, we constructed a new model named model B. The AUC values for LNM prediction between model A and model B was not statistically different (Table 4, Fig. S2). However, the IDI and NRI values showed statistically significant improvement after adding macroscopic type to model A (Table 4), meaning that macroscopic type can also be considered as a risk factor of LNM. Reclassification results of patients who had LNM and those did not have were showed in Table S1 and Table S2.

**Table 4:** Accuracy of the prediction model based on multivariate logistic analysis for estimating the risk of LNM presence.

Variables	Model A	Model B	*P*
**Training set**			
AUC or C-statistics (95% CI)	0.765(0.711–0.814)	0.777(0.724–0.825)	0.263
^**#**^Bias-corrected AUC (95% CI)	0.764(0.702–0.826)	0.778(0.711–0.841)	0.256
IDI, % (95% CI)		1.75(0.23–3.26)	**0.024**
Continuous NRI, % (95% CI)		37.71(12.14–63.27)	**0.004**
**Validation set**			
AUC or C-statistics (95% CI)	0.773(0.705–0.842)	0.790(0.737–0.836)	0.141
^**#**^Bias-corrected AUC (95% CI)	0.772(0.701–0.835)	0.786(0.720–0.855)	0.332
IDI, % (95% CI)		2.12(0.01–4.25)	**0.047**
Continuous NRI, % (95% CI)		45.14(17.82–72.45)	**0.001**

The bold values mean statistical significance.

Model A = tumor size + tumor invasion depth + tumor differentiation + lymphovascular invasion; Model B = tumor size + tumor invasion depth + tumor differentiation + lymphovascular invasion + macroscopic type.

*AUC* area under curve, *IDI* integrated discrimination improvement, *NRI* net re-classification improvement.

^**#**^Bias-corrected AUC: it was calculated by using R software version 4.0.3 with *pROC* package (method = bootstrap, n = 1000).

### Development and validation of a LNM-predicting nomogram

Subsequently, we used ROC analysis to determine the cutoff value of tumor size as 2 cm in training set and 2.5 cm in validation set (Fig. S3). The LNM rates according to the risk factors based on the results of multivariate logistic analysis are summarized in Table S3 and Table S4. Patients with tumors of > 2 cm (training set) or > 2.5 cm (validation set) in size, submucosal invasion, LVI, poor tumor differentiation and non-flat type (I or III) of tumor gross examination seemed to have high LNM rate.

**Figure 2:**
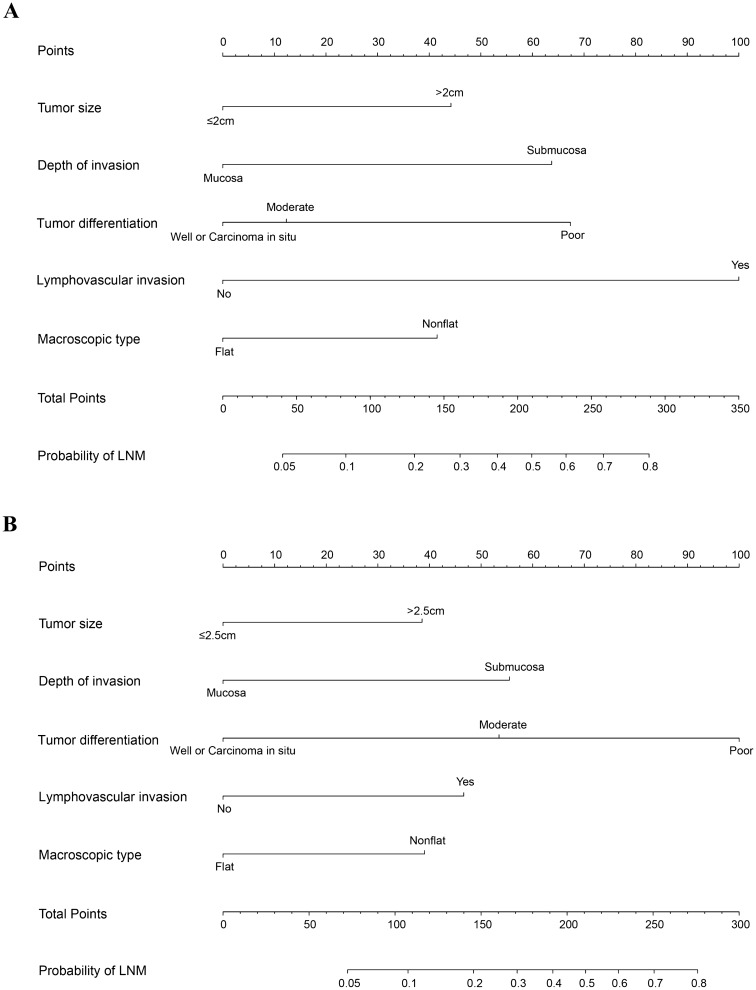
Nomogram for predicting the probability of lymph node metastasis in patients with superficial esophageal squamous cell carcinoma in training set (**A**) and validation set (**B**). Locate the patient’s characteristic on a variable row and draw a vertical line straight up to the points’ row (top) to assign a point value for the variable. Add up the total number of points and drop a vertical line from the total points’ row to obtain the probability of lymph node metastasis.

**Figure 3:**
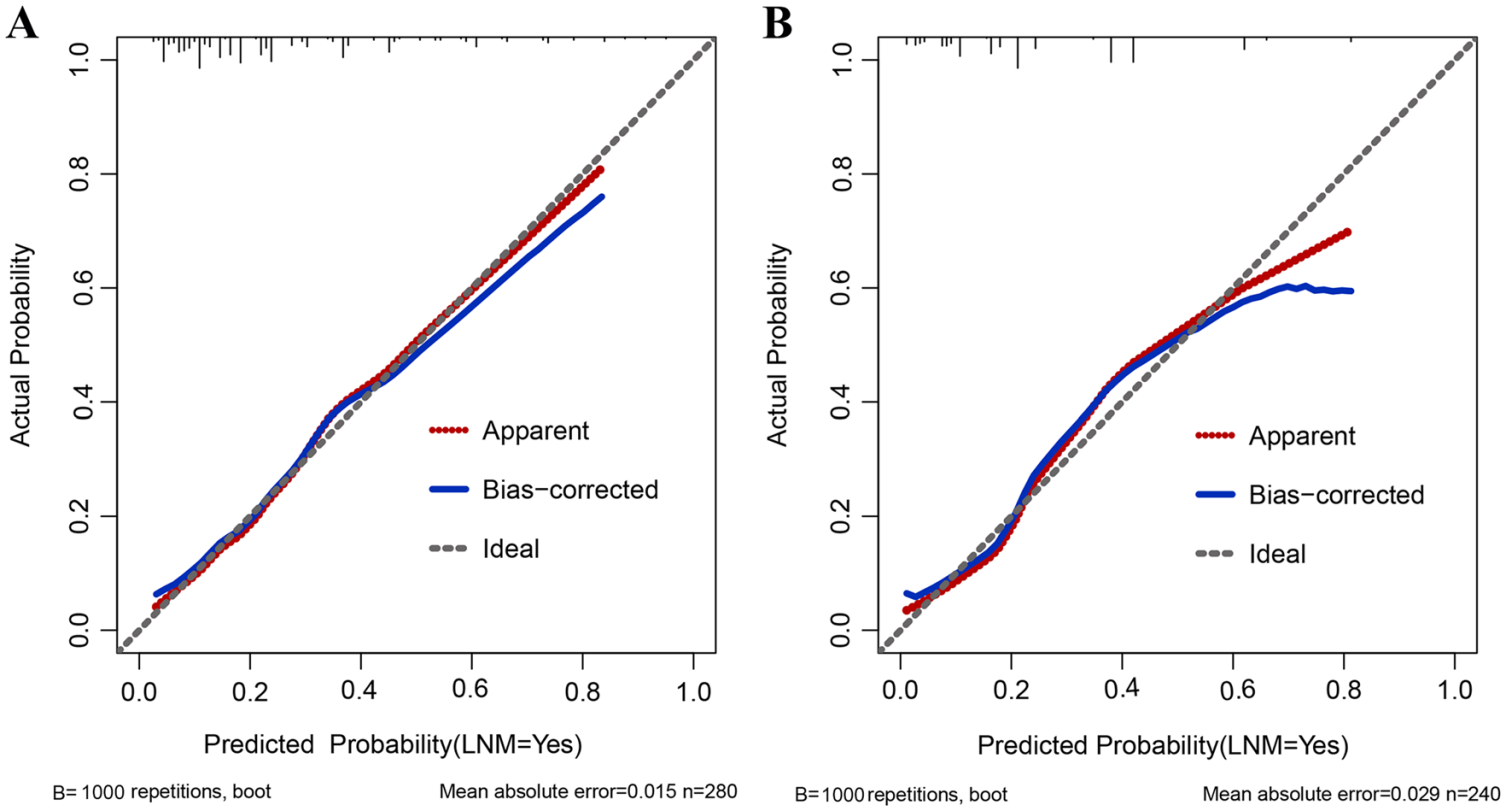
Calibration plot for the nomogram. Validity of the predictive performance of the nomogram in estimating the risk of LNM presence in the training cohort (**A**) and validation set (**B**).

Finally, a nomogram for LNM prediction was formed by incorporating five variables—tumor size, tumor invasion depth, tumor differentiation, LVI and macroscopic type (Fig. 2). The nomogram was validated by internal (bootstrap method) and external validation (validation set). The Hosmer–Lemeshow test yielded a *P* value of 0.995, indicating that the model was well fitted. This nomogram showed a good performance for predicting LNM risk, with an AUC (or C- statistics) of 0.777 (95% CI 0.724–0.825) (Table 4, Fig. S2A) and a bootstrap-corrected AUC of 0.778 (Table 4). Additionally, a calibration curve of the training set demonstrated good consistency between the predicted and observed results regarding the LNM status (Fig. 3A). In validation set, the nomogram achieved an AUC of 0.790 (95% CI 0.737–0.836) for the estimation of LNM risk (Table 4, Fig. S2B), and its calibration curve also fitted well (Fig. 3B).

### The nomogram score system for LNM risk prediction and clinical use

**Figure 4:**
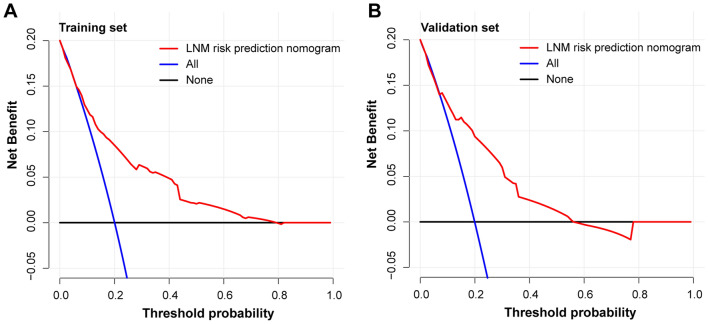
Decision curves of the nomogram predicting LNM in training set (**A**) and validation set (**B**).

Each predictive variable displayed in the nomogram was assigned a risk score. The detailed scores of five variables (tumor size, tumor invasion depth, tumor differentiation, LVI and macroscopic type) in training and validation sets are presented in Fig. 2, Table S5 and Table S6. We predicted the presence of LNM by summing the scores of these five variables, and the final total scores ranged from 0 to 317 in training set and 0 to 281 in validation set. The optimal cutoff points of the total nomogram score for LNM in the training set and validation set were determined to be 150 and 148 respectively according to the ROC curve analysis (Table S7 and Table S8). As a result, patients with total scores ≤ 150 in the training set and ≤ 148 in the validation set were classified as low risk, and patients with total scores of > 150 (the training set) and > 148 (the validation set) were classified as high risk (Table S7 and Table S8). In addition, the DCA in the training and validation sets indicated that our nomogram had significant net benefits for almost all threshold probabilities at different points, suggesting a good clinical utility of this nomogram (Fig. 4).

## Discussion

For the histopathological type of esophageal cancer, adenocarcinoma account for the majority in western countries, while esophageal squamous carcinoma is the predominate type in China^20^. Superficial esophageal squamous cell carcinoma (SESCC) just invade the mucosa and submucosa and lack of any subjective symptoms. Hence, early diagnosis was difficult for these patients, and most esophageal cancers were at a locally advanced stage when the diagnosis was confirmed in the past. However, due to the progress in flexible endoscopic procedure and widespread use of endoscopic screening, the incidence of SESCC is increasing^21^.

In patients of SESCC, LNM contributes a lot to the unfavourable prognosis^22^, resulting in a significantly lower 5-year survival rate in LNM positive patients than in LNM negative patients^23,24^. Endoscopic resection (ER) is mainly suitable for the low-risk LNM patients whose tumors can be completely removed by endoscopic surgery in the light of the guidelines of SESCC diagnosis and treatment^25^. Because of the restriction of ER for lymph node biopsy, we aimed to identify predictors of LNM to prevent them from the presence of tumor cells after ER. Our findings indicated that positive LNM patients were statistically more likely to have larger tumors, poorer differentiation, deeper tumor invasion and LVI in the training and validation sets. Macroscopic type was also determined to be significantly associated with LNM in the multivariate analysis of the validation set, but lost significance in the multivariate analysis of the training set.

Some previous studies reported that LNM seemed to be correlated with tumor size and also these results had statistical significance in SESCC patients^26–29^. It can be concluded from our study that tumor size was significantly correlated with LNM in entire 520 patients and also identified as an important predictor of LNM. Although SESCC comprises both mucosal and submucosal cancers, the LNM status may differ between mucosal and submucosal cancers. Taking mucosal infiltration as reference, the odds ratio of the submucosal infiltration was 3.112 (95% CI 1.025–9.436) for prediction of LNM in our training set (Table 3), demonstrating that the presence of submucosal infiltration was identified as a significant risk factor of LNM. The LNM rate among SESCC patients with mucosal cancer was 8.1% (5/62), while the incidence of LNM increased dramatically to 29.36% (64/218) in patients with submucosal invasion (Table S3). Tumor invasion depth was also reported as a risk factor of LNM in previous studies^6,30,31^, which was similar to our results, suggesting that endoscopic resection might not be appropriate for submucosal cancers^32^.

As well as the tumor invasion depth, LVI was also considered as a remarkable risk factor for LNM in SESCC patients from several studies^31,33,34^. Similarly, it was shown from our data that LVI was significantly related to LNM in SESCC patients (Table 3). For that reason, supplementary surgical therapy with lymph node dissection should be pondered when LVI is detected in the tumor specimen resected by endoscopic surgery. Interestingly, we also found that the LNM rates were still high even in tumors without LVI. For tumors confining to muscularis mucosa with negative LVI, the LNM rates were 6.6% (4/61) and 5.8% (3/52) in training set and validation set respectively; while for the tumors invading to the submucosa without LVI, the LNM rates of training and validation sets increased to 24.2% (46/190) and 26.5% (43/162) respectively (Table S3). Eguchi et al.^31^ pointed out that the LNM rate in SESCC without LVI was 10.3% for tumors involving the muscularis mucosa and was 28.6% for tumors with SM invasion, which is similar to ours. The high rate LNM in SESCC with negative LVI may attribute to the existence of early and skip metastasis along the abundant lymphatic channels in the mucosa and submucosa cancers without LVI. In general, the absence of LVI is also a requirement for curative endoscopic resection.

It was previously reported that histological differentiation was a potential risk factor of LNM^26,28,35^. Consistently, we also found a significant association between tumor differentiation and LNM in the current study (Table 3). Macroscopic appearance of esophageal cancer was seemed to be related to the tumor invasion depth, which might be crucial to evaluate the LNM risk^36^. Interestingly, from our multivariate analysis, there was no correlation between the nonflat type morphology and LNM in training set; in contrast, tumor with nonflat type was identified as an independent risk factor for LNM in validation set (Table 3). Four variables (tumor size, tumor invasion depth, tumor differentiation and LVI) were incorporated to build a model A on the basis of multivariate analyses results. Then model B was further constructed by adding macroscopic type to the model A. Herein, addition of macroscopic type to the model A did not improve AUC values for predicting LNM, but the IDI and NRI values significantly improved (Table 4), indicating that macroscopic type could be considered as a risk factor for LNM.

Moreover, a nomogram was developed for LNM prediction by incorporating the five significant predictors (tumor size, tumor invasion depth, tumor differentiation, LVI and macroscopic type), with an AUC of 0.777 in the training set and 0.790 in the validation set (Table 4, Fig. S2).

The great accuracy and consistency of our nomogram for predicting LNM were confirmed by the calibration curves (Fig. 3). Then the cutoff values of total nomogram score were determined as 150 in training set (Table S7) and 148 in validation set (Table S8) according to the ROC analysis. Patients with a total score of > 150 in the training set and a total score of > 148 in the validation set were considered high-risk, which can guide us to make best treatment decision. Finally, the DCA was performed to confirm the clinical utility of our nomogram and its result showed that if the threshold probability of a patient was > 20%, more benefit was added than either the scheme of treating all patients or the scheme of treating zero patient by using our nomogram to predict LNM (Fig. 4).

In summary, tumor size, tumor invasion depth, tumor differentiation, and LVI were identified as significant predictive factors for LNM in patients with SESCC. Tumor macroscopic type was also identified as a predictor for LNM by calculating the IDI and NRI. Furthermore, a nomogram scoring system was established using these five variables, making individualized LNM prediction easier and facilitating optimal treatment strategy selection for patients with SESCC. Judging from the nomogram scoring system, careful follow-up observation can be recommended if the LNM of SESCC patients after ER is low risk, and supplementary surgery need to be taken if the LNM of SESCC patients after ER is high risk. DCA demonstrated that the nomogram was clinically useful. However, this was a retrospective study based on data from a single institution. Therefore, it is necessary to validate the results using data from multiple centers and a prospective study is required to further confirm the reliability of the nomogram. Last but not least, our nomogram may improve and facilitate treatment strategy selection, which may lead to early diagnosis and prompt treatment initiation for patients with SESCC.

## Supplementary Information

Supplementary Information.

## Abbreviations

SESCC: Superficial esophageal squamous cell carcinoma
LNM: Lymph node metastasis
ER: Endoscopic resection
LVI: Lymphovascular invasion
AUC: Area under the curve
IDI: Integrated discrimination
NRI: Net reclassification improvement
DCA: Decision curve analysis

## References

[1] Bray F (2018). Global cancer statistics 2018: GLOBOCAN estimates of incidence and mortality worldwide for 36 cancers in 185 countries. CA Cancer J. Clin..

[2] Kuwano H (2015). Guidelines for diagnosis and treatment of carcinoma of the esophagus April 2012 edited by the Japan Esophageal Society. Esophagus-Tokyo.

[3] Pech O (2004). Endoscopic resection of superficial esophageal squamous-cell carcinomas: Western experience. Am. J. Gastroenterol..

[4] Roth JA, Putnam JJ (1994). Surgery for cancer of the esophagus. Semin. Oncol..

[5] Mitzman B (2017). Minimally invasive esophagectomy provides equivalent survival to open esophagectomy: An analysis of the national cancer database. Semin, Thorac, Cardiovasc, Surg..

[6] Choi JY (2011). Feasibility of endoscopic resection in superficial esophageal squamous carcinoma. Gastrointest. Endosc..

[7] Ishihara R (2008). Comparison of EMR and endoscopic submucosal dissection for en bloc resection of early esophageal cancers in Japan. Gastrointest. Endosc..

[8] Shi Q (2011). Endoscopic submucosal dissection for treatment of esophageal submucosal tumors originating from the muscularis propria layer. Gastrointest. Endosc..

[9] Kim K, Park SJ, Kim BT, Lee KS, Shim YM (2001). Evaluation of lymph node metastases in squamous cell carcinoma of the esophagus with positron emission tomography. Ann. Thorac. Surg..

[10] Lightdale CJ, Kulkarni KG (2005). Role of endoscopic ultrasonography in the staging and follow-up of esophageal cancer. J. Clin. Oncol..

[11] Yoon YC (2003). Metastasis to regional lymph nodes in patients with esophageal squamous cell carcinoma: CT versus FDG PET for presurgical detection prospective study. Radiology.

[12] The Paris endoscopic classification of superficial neoplastic lesions (2003). Esophagus, stomach, and colon: November 30 to December 1, 2002. Gastrointest. Endosc..

[13] Matsuda S, Takeuchi H, Kawakubo H, Kitagawa Y (2017). Three-field lymph node dissection in esophageal cancer surgery. J. Thorac Dis..

[14] Shimoda T (2011). Japanese classification of esophageal cancer, the 10th edition—pathological part. Nihon Rinsho.

[15] Rice TW, Blackstone EH, Rusch VW (2010). 7Th edition of the AJCC cancer staging manual: Esophagus and esophagogastric junction. Ann. Surg. Oncol..

[16] Pencina MJ, D'Agostino RS, Steyerberg EW (2011). Extensions of net reclassification improvement calculations to measure usefulness of new biomarkers. Stat. Med..

[17] Pencina MJ, D'Agostino RS, D'Agostino RJ, Vasan RS (2008). Evaluating the added predictive ability of a new marker: From area under the ROC curve to reclassification and beyond. Stat. Med..

[18] Steyerberg EW, Vergouwe Y (2014). Towards better clinical prediction models: Seven steps for development and an ABCD for validation. Eur. Heart J..

[19] Collins GS, Reitsma JB, Altman DG, Moons KG (2015). Transparent reporting of a multivariable prediction model for individual prognosis or diagnosis (TRIPOD): The TRIPOD statement. BMJ.

[20] Li B (2013). Prevalence of lymph node metastases in superficial esophageal squamous cell carcinoma. J. Thorac. Cardiovasc. Surg..

[21] The Paris endoscopic classification of superficial neoplastic lesions (2003). Esophagus, stomach, and colon: November 30 to December 1, 2002. Gastrointest. Endosc..

[22] Sabik, J. F. *et al.* Superficial esophageal carcinoma. *Ann. Thorac. Surg.* **60**, 896–901, 902 (1995). 10.1016/0003-4975(95)00542-s 7574991

[23] Bonavina L, Ruol A, Ancona E, Peracchia A (1997). Prognosis of early squamous cell carcinoma of the esophagus after surgical therapy. Dis. Esophagus..

[24] Nabeya K, Nakata Y (1997). Extent of resection and lymphadenectomy in early squamous cell esophageal cancer. Dis. Esophagus..

[25] Ono S (2009). Long-term outcomes of endoscopic submucosal dissection for superficial esophageal squamous cell neoplasms. Gastrointest. Endosc..

[26] Min BH (2020). Nomogram for prediction of lymph node metastasis in patients with superficial esophageal squamous cell carcinoma. J. Gastroen. Hepatol..

[27] Ma DW (2019). Predicting lymph node metastasis for endoscopic resection of superficial esophageal squamous cell carcinoma. J. Thorac. Cardiovasc. Surg..

[28] Zhou Y (2016). Clinicopathologic analysis of lymph node status in superficial esophageal squamous carcinoma. World J. Surg Oncol..

[29] Gockel I (2009). Prediction model of lymph node metastasis in superficial esophageal adenocarcinoma and squamous cell cancer including D2–40 immunostaining. J. Surg. Oncol..

[30] Bollschweiler E (2006). High rate of lymph-node metastasis in submucosal esophageal squamous-cell carcinomas and adenocarcinomas. Endoscopy.

[31] Eguchi T (2006). Histopathological criteria for additional treatment after endoscopic mucosal resection for esophageal cancer: Analysis of 464 surgically resected cases. Mod. Pathol..

[32] Kodama M, Kakegawa T (1998). Treatment of superficial cancer of the esophagus: A summary of responses to a questionnaire on superficial cancer of the esophagus in Japan. Surgery.

[33] Shimada H (2006). Prediction of lymph node status in patients with superficial esophageal carcinoma: Analysis of 160 surgically resected cancers. Am. J. Surg..

[34] Kim DU (2008). Risk factors of lymph node metastasis in T1 esophageal squamous cell carcinoma. J. Gastroenterol. Hepatol..

[35] Pech O (2007). Curative endoscopic therapy in patients with early esophageal squamous-cell carcinoma or high-grade intraepithelial neoplasia. Endoscopy.

[36] Update on the paris classification of superficial neoplastic lesions in the digestive tract. *Endoscopy*.** 37**, 570–578 (2005). 10.1055/s-2005-861352 15933932

