# Supplementary material for: Retrospective analysis of predictive factors for lymph node metastasis in superficial esophageal squamous cell carcinoma
Source: Sci Rep. 2021 Aug 16;11:16544. doi: 10.1038/s41598-021-96088-y (PMC8368005; doi:10.1038/s41598-021-96088-y)
Supplement: Supplementary file 1 — Supplementary Information. [file 41598_2021_96088_MOESM1_ESM.pdf]

Retrospective analysis of predictive factors for lymph node metastasis in superficial esophageal squamous cell carcinoma

Rongwei Ruan†, Shengsen Chen†, Yali Tao†, Jiangping Yu, Danping Zhou, Zhao Cui, Qiwen Shen, Shi Wang*

Department of Endoscopy, Cancer Hospital of the University of Chinese Academy of Sciences(Zhejiang Cancer Hospital), Institute of Cancer and Basic Medicine(IBMC), Chinese Academy of Sciences, Hangzhou 310022, Zhejiang, China.

†Rongwei Ruan, Shengsen Chen and Yali Tao contributed equally to this work.

*Corresponding author: Shi Wang. Department of Endoscopy, Cancer Hospital of the University of Chinese Academy of Sciences(Zhejiang Cancer Hospital), Institute of Cancer and Basic Medicine(IBMC), Chinese Academy of Sciences, Hangzhou 310022, Zhejiang, China.
E-mail: wangshi@zjcc.org.cn, telephone number: +86-571-88122277, fax number: +86-571-88122277.

Figure legends

Figure S1. The prevalence of lymph node metastasis according to the different anatomic site (different lymph node station).

Figure S2. ROC curve to demonstrate the discriminatory ability of model A and model B in predicting the LNM of SESCC patients in training set (A) and validation set (B). Model A= tumor size + tumor invasion depth + tumor differentiation + lymphovascular invasion; Model B= tumor size + tumor invasion depth + tumor differentiation + lymphovascular invasion + macroscopic type.

Figure S3. Determination of the optimal cutoff value for tumor size based on the ROC analysis in training set (A) and validation set (B).

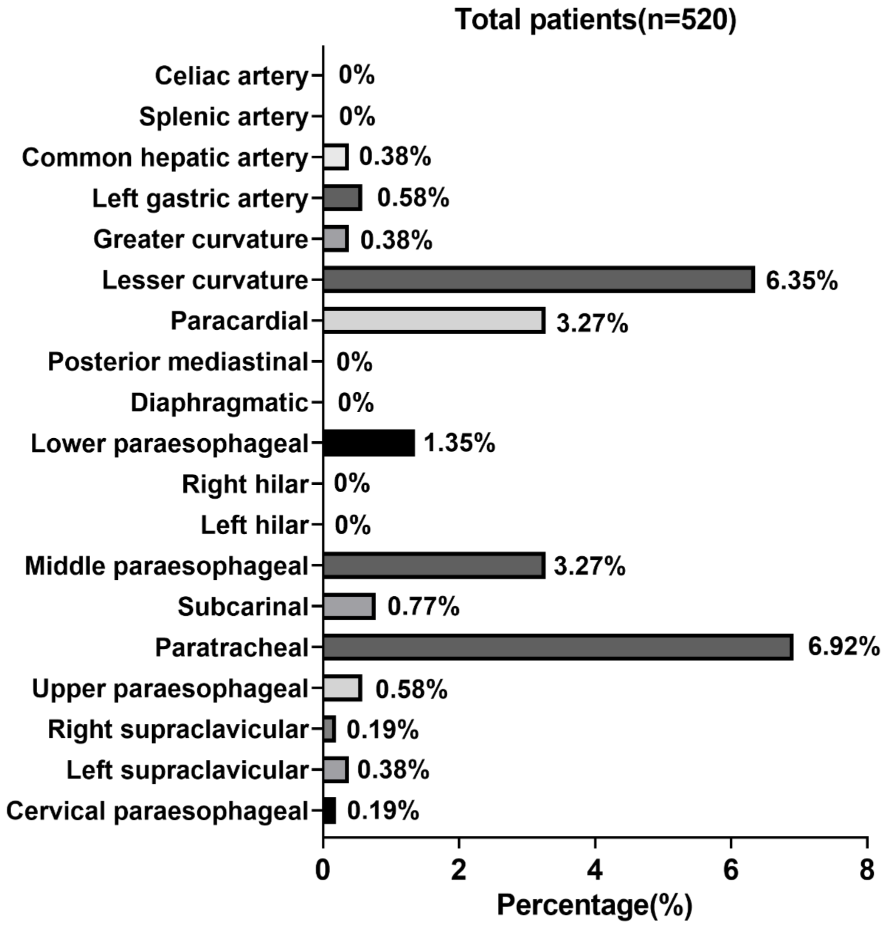
Total patients(n=520)
Celiac artery
Splenic artery
Common hepatic artery
Left gastric artery
Greater curvature
Lesser curvature
Paracardial
Posterior mediastinal
Diaphragmatic
Lower paraesophageal
Right hilar
Left hilar
Middle paraesophageal
Subcarinal
Paratracheal
Upper paraesophageal
Right supraclavicular
Left supraclavicular
Cervical paraesophageal
0%
0%
0.38%
0.58%
0.38%
6.35%
3.27%
0%
0%
1.35%
0%
0%
3.27%
0.77%
6.92%
0.58%
0.19%
0.38%
0.19%
0
2
4
6
8
Percentage(%)

Figure S1

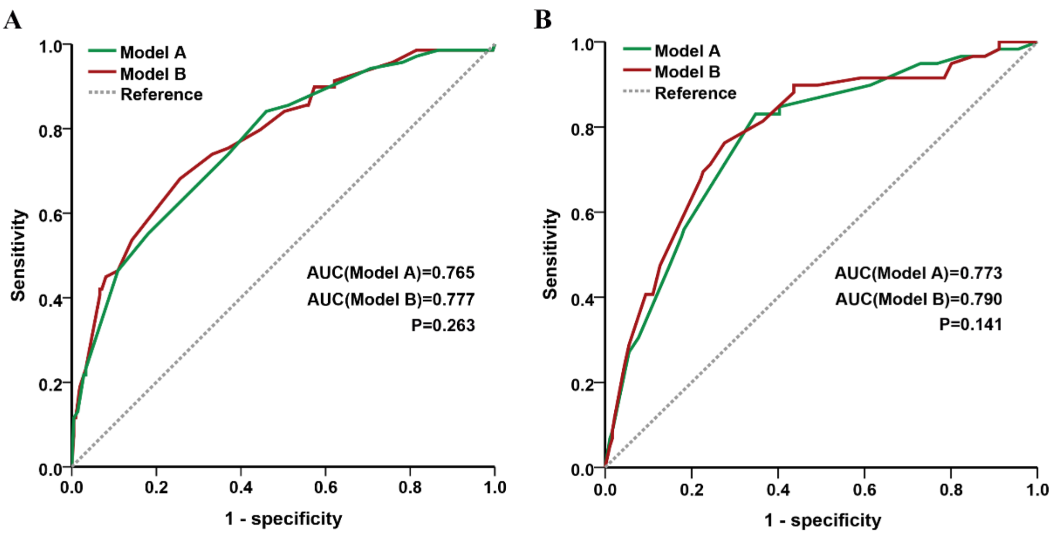

Figure S2

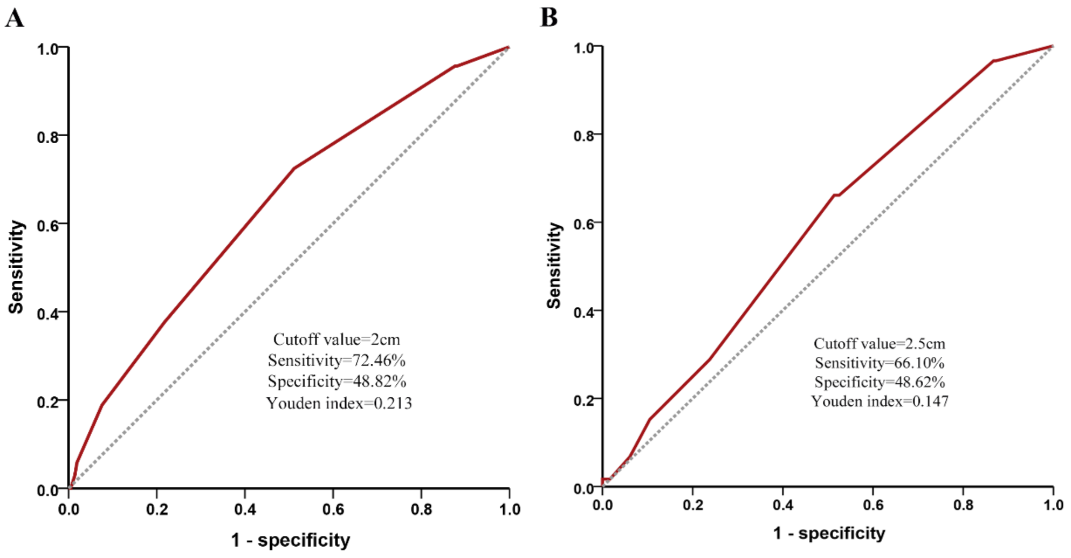

Figure S3

Table S1. Predicted risk of LNM events using a multivariate risk prediction model with and without inclusion of macroscopic type data in training set

Model A (without macroscopic type)	Model B (with macroscopic type)			
Frequency, %	<30	30-60	>60	Total
	Participants who experience a LNM event, n			
<30	22	15	0	37
30-60	0	17	0	17
>60	0	2	13	15
Total	22	34	13	69
	Participants who do not experience a LNM event, n			
<30	157	31	0	188
30-60	0	16	0	16
>60	0	3	4	7
Total	157	50	4	211

Table S2. Predicted risk of LNM events using a multivariate risk prediction model with and without inclusion of macroscopic type data in validation set

Model A (without macroscopic type)	Model B (with macroscopic type)			
Frequency, %	<30	30-60	>60	Total
	Participants who experience a LNM event, n			
<30	10	0	0	10
30-60	8	27	10	45
>60	0	0	4	4
Total	18	27	14	59
	Participants who do not experience a LNM event, n			
<30	118	0	0	118
30-60	22	33	6	61
>60	0	0	2	2
Total	140	33	8	181

TableS3. Lymph node metastasis rates according to tumor size, depth of invasion, and lymphovascular invasion

Tumor size	LVI(-)LNM, n(%)	LVI(+)LNM, n(%)
Training set(n=280)		
Mucosal invasion(n=62)		
≤2cm	2/28(7.1)	0/0(0)
>2cm	2/33(6.1)	1/1(100)
Submucosal invasion(n=218)		
≤2cm	13/85(15.3)	4/9(44.4)
>2cm	33/105(31.4)	14/19(73.7)
Validation set(n=240)		
Mucosal invasion(n=53)		
≤2.5cm	2/23(8.7)	0(0)
>2.5cm	1/29(3.4)	1/1(100)
Submucosal invasion(n=187)		
≤2.5cm	13/74(17.6)	5/11(45.5)
>2.5cm	30/88(34.1)	7/14(50.0)

TableS4. Lymph node metastasis rates according to tumor differentiation and macroscopic type

Tumor differentiation	TypeI LNM, n(%)	TypeII LNM, n(%)	TypeIII LNM, n(%)
Training set(n=280)			
Carcinoma in situ	0/0(0)	1/4(25.0)	0/0(0)
Well	3/27(11.1)	4/21(19.0)	1/3(33.3)
Moderate	17/55(30.9)	9/85(10.6)	3/5(60.0)
Poor	21/38(55.3)	9/41(22.0)	1/1(100)
Validation set(n=240)			
Carcinoma in situ	0(0)	0/12(0)	0(0)
Well	3/26(11.5)	2/14(14.3)	0/1(0)
Moderate	19/49(38.8)	8/64(12.5)	1/8(12.5)
Poor	16/27(59.3)	8/34(23.5)	2/5(40.0)

TableS5. Risk scores based on nomogram for LNM prediction in training set

Factor	Risk scores						
	0	12	42	44	64	67	100
Tumor size(cm)	≤2			>2			
Depth of invasion	Mucosa				Submucosa		
Tumor differentiation	Well or Carcinoma in situ	Moderate				Poor	
LVI	No						Yes
Macroscopic type	Flat		Nonflat				

TableS6. Risk scores based on nomogram for LNM prediction in validation set

Factor	Risk scores				
	0	39	47	53	56	100
Tumor size(cm)	≤2.5	>2.5				
Depth of invasion	Mucosa				Submucosa	
Tumor differentiation	Well or Carcinoma in situ			Moderate		Poor
LVI	No		Yes			
Macroscopic type	Flat	Nonflat				

TableS7. Identification of the optimal cutoff value of the total nomogram scores in ROC curve in training set

Criterion	Sensitivity%	95% CI	Specificity%	95% CI	Youden index
≥0	100.00	94.8 - 100.0	0.00	0.0 - 1.7	0
>0	98.55	92.2 - 100.0	0.47	0.01 - 2.6	-0.0098
>12	98.55	92.2 - 100.0	8.53	5.1 - 13.1	0.0708
>44	98.55	92.2 - 100.0	12.80	8.6 - 18.1	0.1135
>54	98.55	92.2 - 100.0	13.27	9.0 - 18.6	0.1182
>56	98.55	92.2 - 100.0	18.48	13.5 - 24.4	0.1703
>64	97.10	89.9 - 99.6	21.33	16.0 - 27.5	0.1843
>67	95.65	87.8 - 99.1	24.17	18.6 - 30.5	0.1982
>76	91.30	82.0 - 96.7	37.91	31.3 - 44.8	0.2921
>98	89.86	80.2 - 95.8	37.91	31.3 - 44.8	0.2777
>106	89.86	80.2 - 95.8	42.65	35.9 - 49.6	0.3251
>108	85.51	75.0 - 92.8	44.08	37.3 - 51.1	0.2959
>109	85.51	75.0 - 92.8	44.55	37.7 - 51.5	0.3006
>111	84.06	73.3 - 91.8	49.76	42.8 - 56.7	0.3382
>118	79.71	68.3 - 88.4	55.45	48.5 - 62.3	0.3516
>120	75.36	63.5 - 84.9	63.03	56.1 - 69.6	0.3839
>131	73.91	61.9 - 83.7	66.82	60.0 - 73.1	0.4073
**>150**	**68.12**	**55.8 - 78.8**	**74.41**	**68.0 - 80.2**	**0.4253**
>162	53.62	41.2 - 65.7	85.78	80.3 - 90.2	0.394
>173	46.38	34.3 - 58.8	89.10	84.1 - 93.0	0.3548
>175	44.93	32.9 - 57.4	91.94	87.4 - 95.2	0.3687
>176	42.03	30.2 - 54.5	92.89	88.5 - 96.0	0.3492
>208	42.03	30.2 - 54.5	93.36	89.1 - 96.3	0.3539
>211	40.58	28.9 - 53.1	93.36	89.1 - 96.3	0.3394
>217	23.19	13.9 - 34.9	96.68	93.3 - 98.7	0.1987
>218	21.74	12.7 - 33.3	97.16	93.9 - 98.9	0.189
>220	20.29	11.6 - 31.7	97.63	94.6 - 99.2	0.1792
>231	18.84	10.4 - 30.1	98.10	95.2 - 99.5	0.1694
>262	11.59	5.1 - 21.6	99.05	96.6 - 99.9	0.1064
>273	11.59	5.1 - 21.6	99.53	97.4 - 100.0	0.1112
>275	7.25	2.4 - 16.1	99.53	97.4 - 100.0	0.0678
>317	0.00	0.0 - 5.2	100.00	98.3 - 100.0	0

TableS8. Identification of the optimal cutoff value of the total nomogram scores in the ROC curve in validation set

Criterion	Sensitivity%	95% CI	Specificity%	95% CI	Youden index
≥0	100.00	93.9 - 100.0	0.00	0.0 - 2.0	0
>0	100.00	93.9 - 100.0	4.42	1.9 - 8.5	0.0442
>39	98.31	90.9 - 100.0	8.84	5.1 - 14.0	0.0715
>53	96.61	88.3 - 99.6	12.15	7.8 - 17.8	0.0876
>56	96.61	88.3 - 99.6	14.92	10.1 - 21.0	0.1153
>92	94.92	85.9 - 98.9	19.89	14.3 - 26.5	0.1481
>95	91.53	81.3 - 97.2	24.31	18.3 - 31.2	0.1584
>100	91.53	81.3 - 97.2	28.18	21.8 - 35.3	0.1971
>109	91.53	81.3 - 97.2	40.33	33.1 - 47.9	0.3186
>131	91.53	81.3 - 97.2	40.88	33.6 - 48.4	0.3241
>134	89.83	79.2 - 96.2	50.83	43.3 - 58.3	0.4066
>139	88.14	77.1 - 95.1	56.35	48.8 - 63.7	0.4449
**>148**	**76.27**	**63.4 - 86.4**	**72.38**	**65.3 - 78.7**	**0.4865**
>156	69.49	56.1 - 80.8	77.35	70.6 - 83.2	0.4684
>181	67.80	54.4 - 79.4	77.90	71.1 - 83.7	0.457
>187	47.46	34.3 - 60.9	87.29	81.5 - 91.8	0.3475
>195	23.73	13.6 - 36.6	95.58	91.5 - 98.1	0.1931
>234	8.47	2.8 - 18.7	98.34	95.2 - 99.7	0.0681
>242	5.08	1.1 - 14.1	98.90	96.1 - 99.9	0.0398
>281	0.00	0.0 - 6.1	100.00	98.0 - 100.0	0
